# Sedimentological constraints on the initial uplift of the West Bogda Mountains in Mid-Permian

**DOI:** 10.1038/s41598-018-19856-3

**Published:** 2018-01-23

**Authors:** Jian Wang, Ying-chang Cao, Xin-tong Wang, Ke-yu Liu, Zhu-kun Wang, Qi-song Xu

**Affiliations:** 1 School of Geosciences, China University of Petroleum (East China), Qingdao, 266580 China; 2 Laboratory for Marine Mineral Resources, Qingdao National Laboratory for Marine Science and Technology, Qingdao, 266071 China; 3 Department of Applied Geology, Curtin University, GPO Box U1987, Perth, WA 6845 Australia

## Abstract

The Late Paleozoic is considered to be an important stage in the evolution of the Central Asian Orogenic Belt (CAOB). The Bogda Mountains, a northeastern branch of the Tianshan Mountains, record the complete Paleozoic history of the Tianshan orogenic belt. The tectonic and sedimentary evolution of the west Bogda area and the timing of initial uplift of the West Bogda Mountains were investigated based on detailed sedimentological study of outcrops, including lithology, sedimentary structures, rock and isotopic compositions and paleocurrent directions. At the end of the Early Permian, the West Bogda Trough was closed and an island arc was formed. The sedimentary and subsidence center of the Middle Permian inherited that of the Early Permian. The west Bogda area became an inherited catchment area, and developed a widespread shallow, deep and then shallow lacustrine succession during the Mid-Permian. At the end of the Mid-Permian, strong intracontinental collision caused the initial uplift of the West Bogda Mountains. Sedimentological evidence further confirmed that the West Bogda Mountains was a rift basin in the Carboniferous-Early Permian, and subsequently entered the Late Paleozoic large-scale intracontinental orogeny in the region.

## Introduction

**Figure 1:**
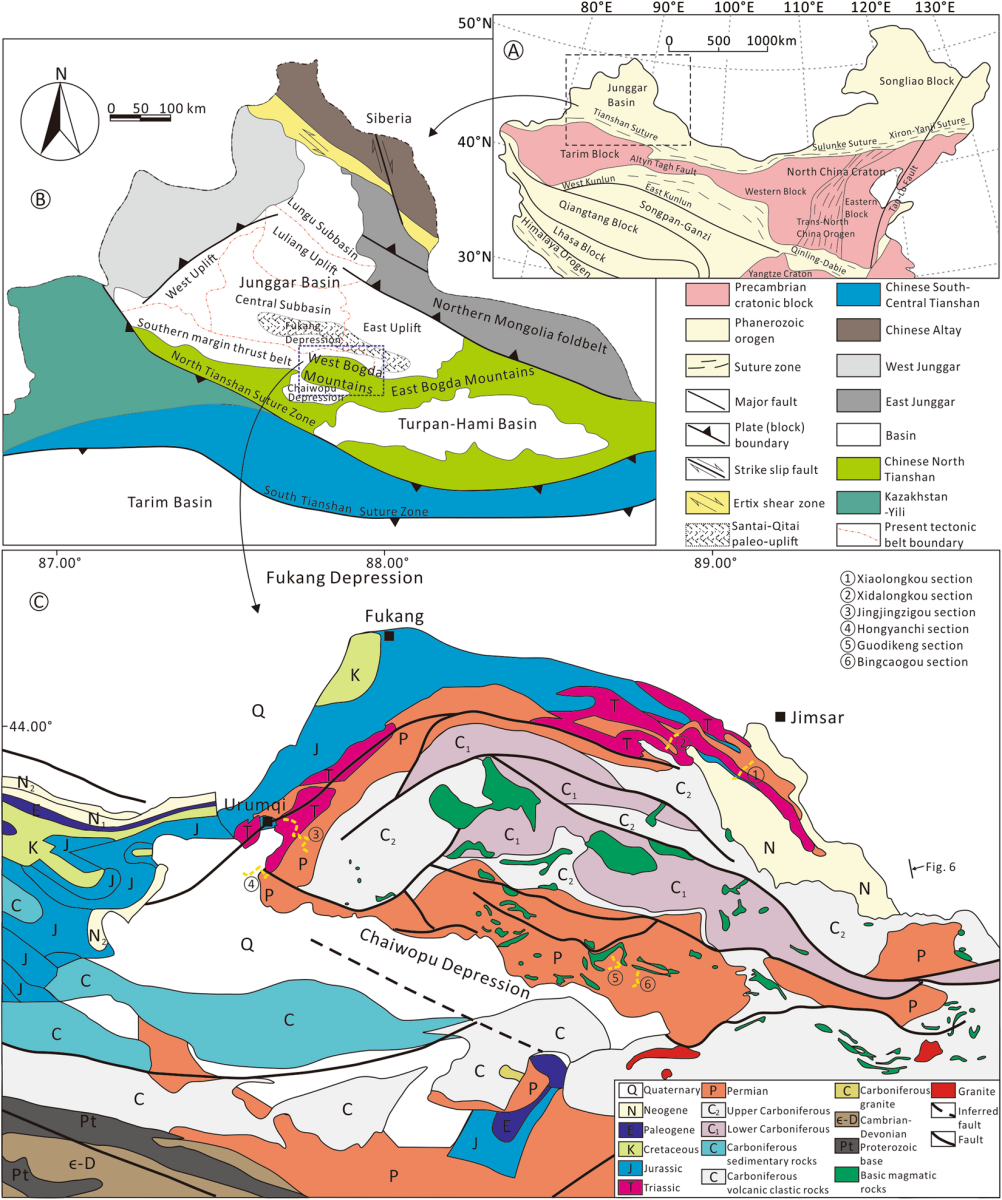
(**A**) Tectonic map of North China. (**B**) Tectonic map of Northwest China, present tectonic belts of the Junggar Basin and the tectonic setting of the study area. (**C**) Distribution of strata around the West Bogda Mountains and locations of the outcrop sections. The area of the Santai-Qitai paleo-uplift is modified from Lin^38^, which was subsided during the Middle Permian. The maps were generated by GeoMap 3.6 (http://jurassic.com.cn:8080/english/) and CorelDraw X7 (http://www.corel.com/cn/).

The Central Asia Orogenic Belt (CAOB) is the largest accretionary orogen on Earth, which was formed by the amalgamation of multiple micro-continents, island arcs and accretionary wedges^1–5^. The Late Carboniferous-Permian period is considered to be an important turning point of the Late Paleozoic amalgamation of continents and Mesozoic-Cenozoic intracontinental basin-mountain system of CAOB, and thus an important stage in its evolution^3,4,6–9^. As the continental interior mountain chain is far from plate boundaries, the Tianshan Mountains not only play an important role in the Paleozoic subduction collision zone, but also has been subjected to intense compression and uplift and other tectonic deformation since the Cenozoic era, forming a typical rejuvenated orogenic belt^2,3,6,9^. The Tianshan orogenic belt in northwestern China is an important component and key for understanding the Paleozoic evolution of CAOB^3,9^. The Bogda Mountains are located in the northern region of the eastern part in the Tianshan Mountains and become an integral part of the Tianshan orogenic belt, comprising the West Bogda Mountains and the East Bogda Mountains (Fig. 1B)^10^. The West Bogda Mountains adjacent to the north Junggar Basin and south Turpan-Hami Basin, is an ideal place to study the kinematics and dynamics of the Tianshan orogenic belt. The tectonic attribute of the Late Paleozoic intracontinental orogenic belt of the West Bogda Mountains remains a hot and controversial issue at present^10–18^, despite being intensively studied. There is no consensus on some key issues, especially the timing of the initial uplift^10–18^. At present, there are four main viewpoints proposed: (1) The first large-scale uplift of the West Bogda Mountains was during the Middle-Late Jurassic, followed by three stages of large-scale uplifts in the Early Paleocene, Middle Eocene and Late Oligocene based on apatite fission track (AFT) and detrital zircon U-Pb dating^13,16,19,20^; (2) According to AFT and detrital zircon U-Pb dating, and paleocurrent direction analysis, the Bogda Mountains and its adjacent areas were thought to be initially uplifted in the Early Permian from a remnant ocean trough^11,12,15^, followed by two large-scale uplift events in the Late Jurassic and Cenozoic^12,17,21–23^; (3) The initial uplift of the West Bogda Mountains was considered to have occurred in the Late Permian-Early Triassic on the basis of the structural attributes of the Carboniferous volcanic rocks, the development of ductile shear belts and stratigraphic contact relationships within the Tianshan orogenic belt^14,15,18^; (4) The initial uplift of the West Bogda Mountains was during the Middle Triassic, followed by two subsequent large-scale uplift events in the Late Jurassic-Early Cretaceous and Cenozoic based on the characteristics of multiple stages of folds in the Bogda Mountains^10^. The initial uplift timing of the West Bogda Mountain is therefore a fundamental geological problem involving the tectonic attribute of the Bogda Mountains, structural transformation of the Junggar Basin and the Paleozoic orogenic belt range of the Tianshan Mountains^10,12,13^. Previous studies on the uplift timing and process of the West Bogda Mountains were mainly based on petrology, petrogeochemistry and thermal geochronology of the widely distributed volcanic rocks and pyroclastic rocks. The sediment sequences also contain a great deal of geodynamic information on the orogenic evolution and can record important sedimentary responses to orogenic processes^10,16,24–26^. Basin filling successions provide the most continuous and sensitive geological records reflecting the evolution of adjacent orogenic belts^27^. Strong orogeny usually causes rapid changes or discontinuity of stratigraphic record^16,28^. For example, an in-depth study of orogenic belts of the North American Laramide and Cordilleran^29^ and the Asian Himalaya^30^, and other cases^16,24–26,31^ had revealed the intimate relationship between sedimentation and tectonic environment, the relationship between terrigenous deposits and regional tectonic evolution. Based on a detailed sedimentological study of outcrops in the West Bogda Mountains and combined with previous work on the regional tectonic evolution, we attempt to determine the timing of initial uplift of the West Bogda Mountains, which may have important significance in understanding the evolution of the Tianshan orogenic belt.

The Bogda Mountains, a northeastern branch of the Tianshan Mountains, are located in the southern part of the Central Asia Orogenic Belt and the southeastern margin of the Junggar Basin (Fig. 1A). The Bogda Mountains extend more than 250 kilometers in an E-W direction, and exhibit northward arc-shape ranges that separate the southern Turpan-Hami Basin and the northern Junggar Basin (Fig. 1B). The West Bogda Mountains are located between the northern Fukang depression and the southern Chaiwopu depression (Fig. 1C). The main body of the West Bogda Mountains developed in the Carboniferous-Early Permian with bimodal volcanic-sedimentary rock series (342 Ma-278 Ma)^11,15,32^, while the north and south margins of the foothills comprise Upper Paleozoic, Mesozoic, and Cenozoic sedimentary strata (Fig. 1C).

**Figure 2:**
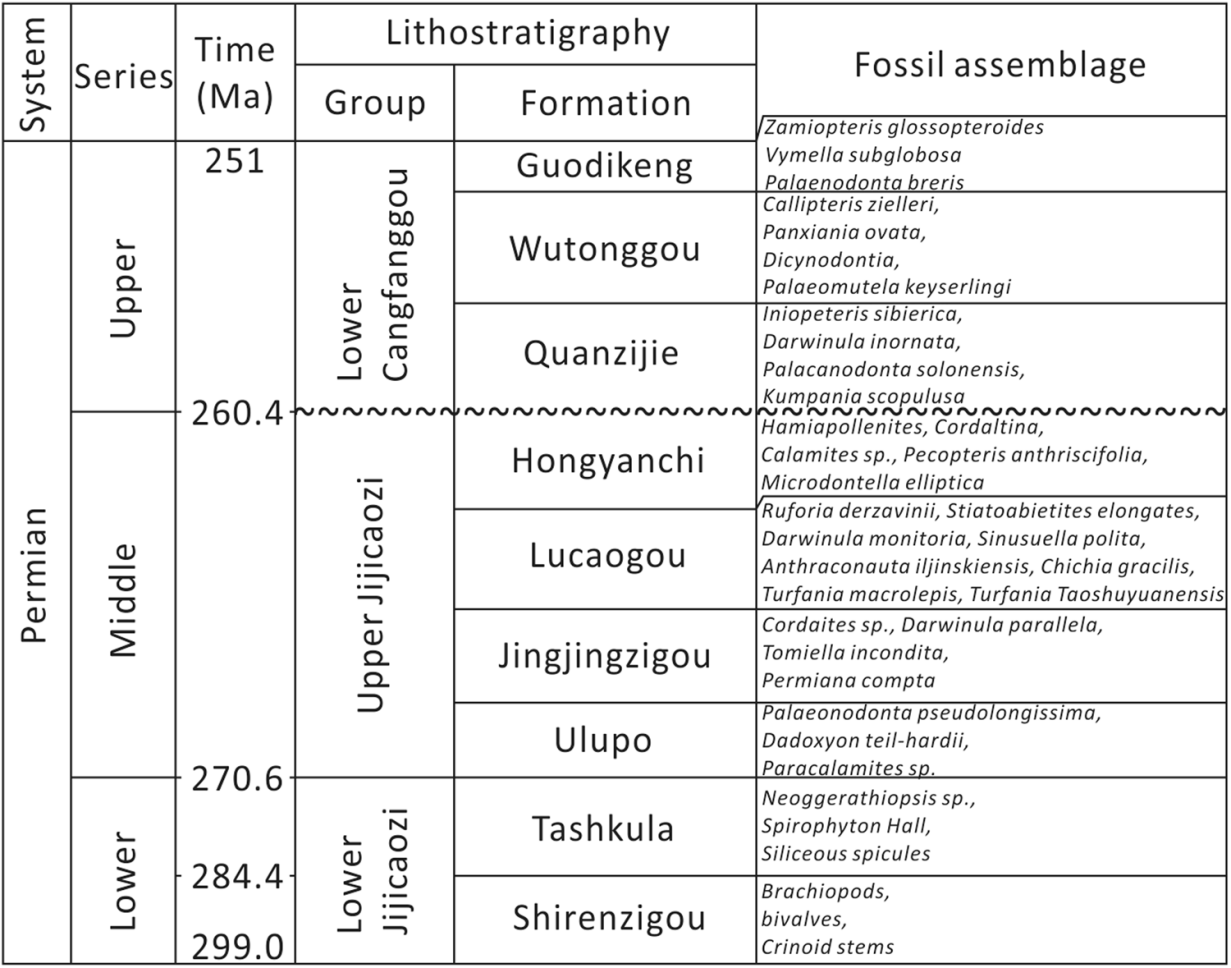
Chrono- and lithostratigraphy of the Permian strata around the West Bogda Mountains. Wavy lines mark major unconformities. Fossils are from Wartes *et al*.^33^. Absolute ages are from Yang *et al*.^37^.

The northern and western margins of the West Bogda Mountains comprise Upper Paleozoic (Permian), Mesozoic (Triassic, Jurassic and Cretaceous) and Cenozoic formations, while the southern margin is composed mainly of the Upper Paleozoic (Permian) formations (Fig. 1C). The Permian stratigraphic sequence is generally divided into the Lower Jijicaozi Group, the Upper Jijicaozi Group and the Lower Cangfanggou Group, corresponding to the Early Permian, Middle Permian and Late Permian, respectively (Fig. 2). The fossil assemblages of the formations are shown in the Fig. 2^33^. The fossils in the Shirenzigou and Tashkula formations consist mainly of marine *Brachiopods*, *Bivalves*, *Crinoid stems*, *Spirophyton Hall* and *Siliceous spicules*, while fossils in the overlying Middle and Upper Permian strata comprise mainly nonmarine Fauna (*Sinusuella polita*, *Chichia gracilis*, *Urumqia liduaowanensis*, *Dicynodontia*, etc.) and Flora (*Neoggerathiopsis sp*., *Cordaianthus volkmannii*, *Paracalamites sp*., *Cordaites sp*., *Hamiapollenites*, *Cordaltina*, *Pecopteris anthriscifolia*, etc.) and freshwater bivalves (*Palaeonodonta pseudolongissima*, *Anthraconauta iljinskiensis*, *Microdontella elliptica*, etc.)^33^. The Permian strata crop out most completely around the Urumqi area with the exposure degrading gradually to the East. The Upper Permian predominantly crops out near Jimsar, while the Lower Permian mainly crops out at the southern margin.

## Results

### Stratigraphic sedimentary characteristics

**Figure 3:**
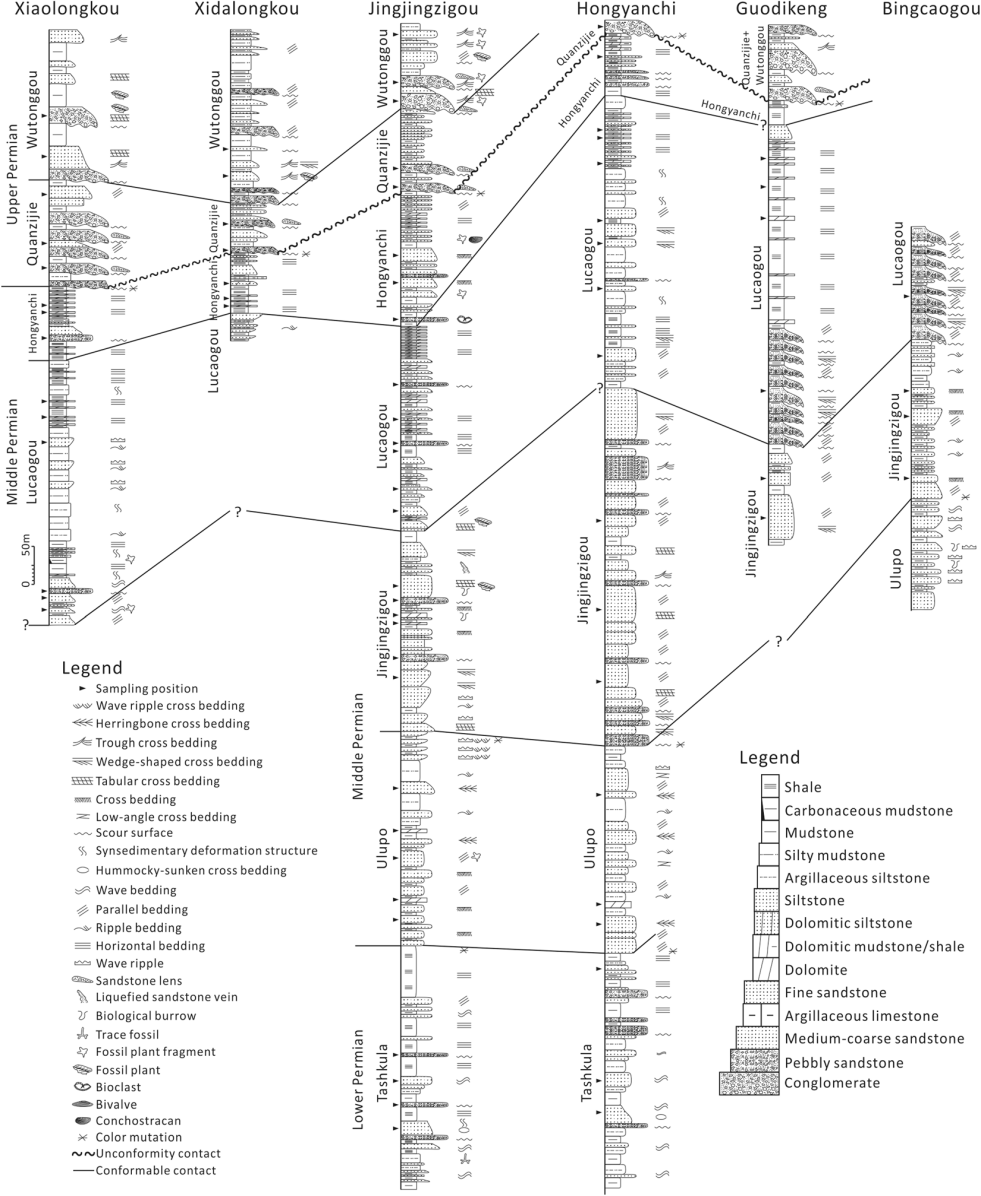
Simplified lithofacies and sedimentary structures, and stratigraphic correlation of outcrop sections around the West Bogda Mountains. The bottom of the Lucaogou Formation in the Xiaolongkou section is uncertain because of poor exposure. Influenced by the geological structures and surface topography, the Lucaogou Formation and underlying formations in the Xidalongkou section, the Jingjingzigou Formation and underlying formations in the Guodikeng section, the Locaogou Formation and overlying formations and the Ulupo Formation and underlying formations in the Bingcaogou section are not exposed. See Fig. 1C for locations of sections.

The Tashkula Formation crops out in the Jingjingzigou section and Hongyanchi section (Fig. 3). The bottom of the Tashkula Formation mainly contains interlayers of gray mudstone, argillaceous siltstone and thin layers of sandstone with wavy bedding and trace fossil (Fig. 3 and Supplementary Fig. S1A). The middle part of the Tashkula Formation mainly occurs as normally graded pebbly sandstone to fine sandstone, argillaceous siltstone or mudstone with scouring surface, hummocky-sunken cross bedding and wavy bedding (Fig. 3 and Supplementary Fig. S1B), upward transitioning to frequently interbedded thin layers of gray siltstone, argillaceous siltstone and mudstone (Fig. 3). The upper part of the Tashkula Formation consists mainly of thick layers of gray-dark mudstone interbedded with thin layers of gray siltstone and pebbly sandstone with modest sorting and rounded pebbles (Fig. 3 and Supplementary Fig. S1C).

The Ulupo Formation, mainly cropping out in the Jingjingzigou section, Hongyanchi section and Bingcaogou section (Fig. 3), is in conformable contact with the underlying Tashkula Formation. The lower part of the Ulupo Formation mainly occurs as interbedded thin layers of gray-greenish siltstone and thick layers of gray fine sandstone with herringbone cross bedding and parallel bedding (Fig. 3). The middle part of the Ulupo Formation is characterized by thick layers of gray argillaceous siltstone interbedded with medium-thin layers of gray-green normally graded siltstone with herringbone cross bedding, ripple bedding, low-angle cross bedding and plant clast (Fig. 3 and Supplementary Fig. S1D). Medium-thin layers of dolomite occur in the lower and middle parts of the Ulupo Formation (Fig. 3). The upper part of the Ulupo Formation develops moderately thick layers of gray-green and reddish silty mudstone interbedded with gray-greenish fine sandstone with wave ripple, wave ripple cross bedding and herringbone cross bedding (Fig. 3 and Supplementary Fig. S1E–F).

The Jingjingzigou Formation, cropping mainly out in the Jingjingzigou section, Hongyanchi section, Guodikeng section and Bingcaogou section, is in conformable contact with the underlying Ulupo Formation (Fig. 3). The lower part of the Jingjingzigou Formation is characterized by normally graded cycles of pebbly sandstone-fine sandstone-siltstone with scouring surface, wedge-shaped cross bedding, tabular cross bedding and parallel bedding (Fig. 3 and Supplementary Fig. S1G). Gravels in pebbly sandstones are mainly of igneous rocks with modest sorting and roundness. Thick layers of inversely graded sandstone can be seen locally with wave ripple and cross bedding (Fig. 3 and Supplementary Fig. S1H). The middle part of the Jingjingzigou Formation occur mainly as thick layers of gray-greenish pebbly sandstone, to medium-fine sandstone, siltstone and mudstone with interlayers of thin dolomite (Fig. 3). Trough cross bedding, tabular cross bedding, biological burrows and plant clasts are common in the sandstones (Fig. 3). The upper part of the Jingjingzigou Formation is mainly characterized by thick layers of pebbly sandstone and coarse-medium sandstone with thin interlayers of mudstone with trough cross bedding, tabular cross bedding, wedge-shaped cross bedding and plant clast (Fig. 3).

The Lucaogou Formation occurs in all six sections and is in conformable contact with the underlying Jingjingzigou Formation (Fig. 3). The lithologies of the north and south sides of the West Bogda Mountains are quite different. At the northern side of the West Bogda Mountains, the lower part of the Lucaogou Formation is dominated by normally graded cycles of medium-thick layers of gray-greenish fine sandstone-gray mudstone with parallel bedding and tabular cross bedding, while the upper part of the Lucaogou Formation is characterized by thick layers of gray-dark shale with thin-medium interlayers of dolomitic sandstone and dolomite (Fig. 3 and Supplementary Fig. S2A–D). At the southern side of the West Bogda Mountains, the Lucaogou Formation is characterized by multiple thick layers of normally graded cycles of gray pebbly sandstone-fine sandstone-gray-dark mudstone (Fig. 3 and Supplementary Fig. S2E–H). At the bottom of the cycles, the pebbly sandstone with wedge-shaped cross bedding and parallel bedding exhibits scouring contacts with the underlying sandstone or mudstone. Gravels are dominated by igneous rocks with modest sorting and roundness (Supplementary Fig. S2H). The upper part also develops thick layers of gray-dark shale with medium-thin layers of dolomite (Fig. 3).

The Hongyanchi Formation is present in all six sections and is in conformable contact with the underlying Lucaogou Formation (Fig. 3). The sedimentary characteristics of the lower part of the Hongyanchi Formation is similar to that of the upper part of the Lucaogou Formation at the northern side of the West Bogda Mountains, which is dominated by thick layers of gray-dark shale with thin interlayers of dolomitic siltstone, siltstone and pebbly sandstone (Fig. 3 and Supplementary Fig. S2I–J). The upper part of the Hongyanchi Formation is characterized by medium-thick layers of gray mudstone with thin layers of gray-greenish fine sandstone (Fig. 3). The upper part of the Hongyanchi Formation had been eroded away in many sections (Fig. 3).

The Quanzijie Formation is in unconformable contact with the underlying Hongyanchi Formation, and is dominated by purplish-reddish conglomerate, pebbly sandstone, sandstone and siltstone. Across the formation boundary, the colors of the rocks change from gray to purplish red (Fig. 3 and Supplementary Fig. S3E, G). Dolomite, dolomitic sandstone and the gray-dark shale gravels are often found in the conglomerate with extremely poor sorting and roundness (Supplementary Fig. S3A–G).

The Wutonggou Formation is in conformable contact with the underlying Quanzijie Formation, and is also characterized by purplish-reddish conglomerate, pebbly sandstone, sandstone and siltstone. The sorting and roundness of gravels are extremely poor, and dolomite, dolomitic sandstone gravels are commonly present (Fig. 3 and Supplementary Fig. S3H–I). A variety of sedimentary structures occur in conglomerate, pebbly sandstone and sandstone including large-scale trough cross bedding, wedge-shaped cross bedding, tabular cross bedding and parallel bedding and plant fragments (Fig. 3).

### Characteristics of rock compositions

The type and amounts of gravels in conglomerate and pebbly sandstone of the Permian strata from the Xiaolongkou and Jingjingzigou sections are listed in Table 1. Gravels of the Permian strata are mainly of igneous rock, tuff, vein quartz, sandstone, siltstone (includes siltstone and dolomitic siltstone), mudstone (includes mudstone, gray-dark shale, and dolomitic shale), and dolomite. Gravel types and amounts in the Early Permian Tashkula Formation, Middle Permian Ulupo and Jingjingzigou Formations and Middle Permian Lucaogou and Hongyanchi Formations are similar. They are dominated by igneous rock components (Table 1). The Late Permian Quanzijie and Wutonggou Formations contain similar gravel types and amounts, which are characterized by high contents of sandstone-siltstone, mudstone and tuff gravels (Table 1). Compared with the Lower and Middle Permian strata, the gravels of the Upper Permian strata are characterized by low igneous rock content but relatively high contents of sandstone-siltstone, mudstone, tuff and dolomite (Table 1). The Wutonggou Formation contains more igneous rock and tuff gravels than that of the Quanzijie Formation, but less sedimentary gravels (Table 1).

**Table 1:** Types and abundances of gravels in conglomerates and pebbly sandstones of the Permian strata from the Xiaolongkou and Jingjingzigou outcrop sections.

Section	Formation	Igneous rock		Tuff		Vein quartz		Sandstone & siltstone		Mudstone		Dolomite		Sum
		No.	%	No.	%	No.	%	No.	%	No.	%	No.	%	
Xiaolongkou	Lucaogou and Hongyanchi	115	69.3	16	9.6	7	4.2	22	13.3	6	3.6	0	0	166
	Quanzijie	36	8.3	121	27.9	30	6.9	179	41.2	43	9.9	25	5.8	434
	Wutonggou	85	17.4	158	32.3	43	8.8	164	33.5	25	5.1	14	2.9	489
Jingjingzigou	Tashkula	161	78.2	10	4.9	4	1.9	24	11.7	6	2.9	1	0.5	206
	Ulupo and Jingjingzigou	224	67.5	40	12	13	3.9	48	14.5	7	2.1	0	0	332
	Lucaogou and Hongyanchi	126	71.6	17	9.7	6	3.4	20	11.4	7	4	0	0	176
	Quanzijie	48	9.3	122	23.6	34	6.6	231	44.6	60	11.6	23	4.4	518
	Wutonggou	80	15.8	162	32	46	9.1	170	33.5	32	6.3	17	3.4	507

**Figure 4:**
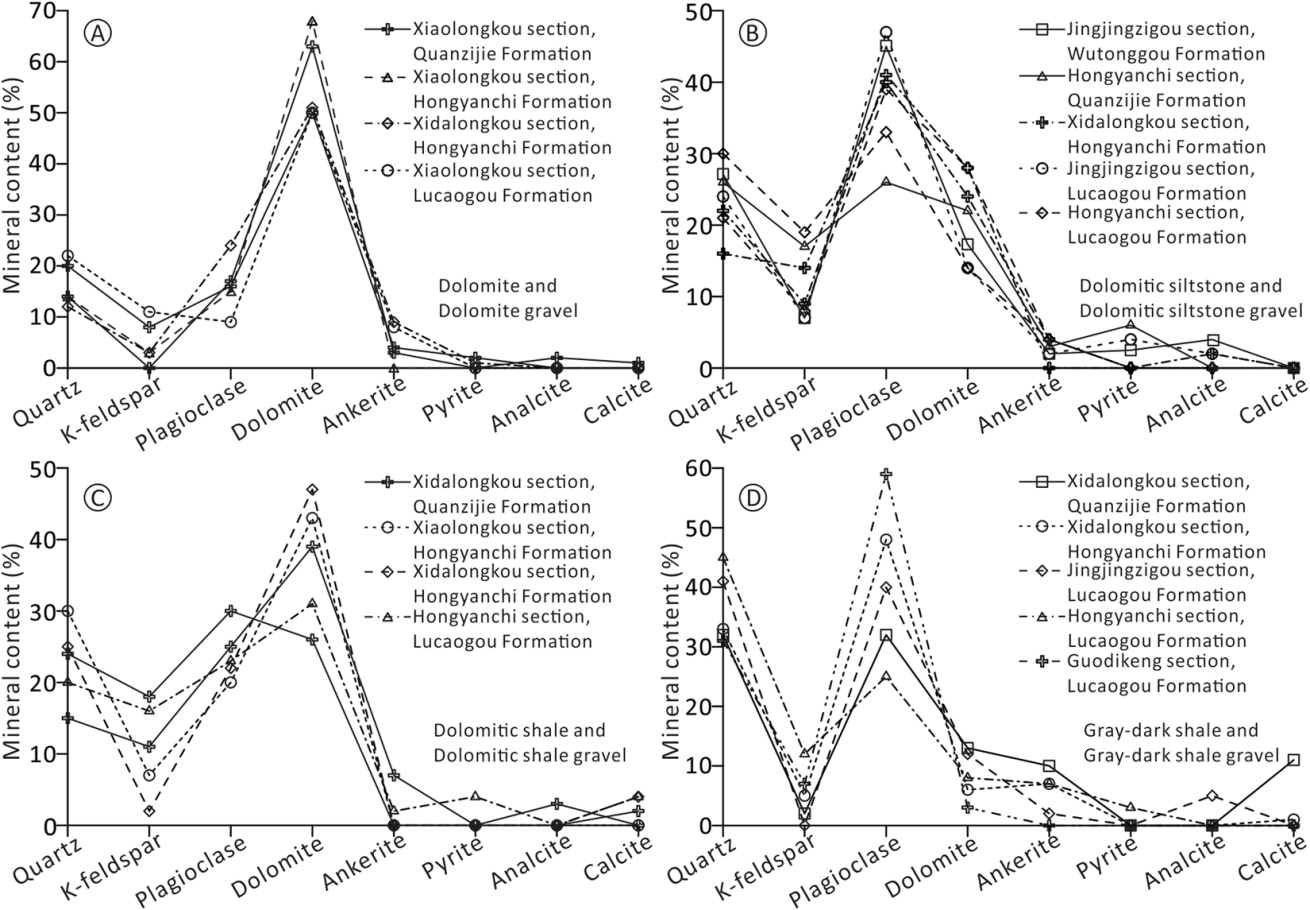
Distribution of the main minerals in dolomite, dolomitic siltstone, dolomitic shale and dark-gray shale of the Lucaogou and Hongyanchi Formations and the corresponding gravels of the Quanzijie and Wutonggou Formations from different sections around the West Bogda Mountains.

XRD analysis indicates that the mineral compositions of dolomite, dolomitic siltstone, dolomitic shale and gray-dark shale of the Lucaogou and Hongyanchi formations in different sections have similar contents and distribution characteristics (Fig. 4). The mineral composition and the distribution of dolomite gravels, dolomitic siltstone gravels, dolomitic shale gravels and gray-dark shale gravels of the Quanzijie or Wutonggou formations are similar to the mineral compositions and distribution of dolomite, dolomitic siltstone, dolomitic shale and gray-dark shale of the Lucaogou and Hongyanchi formations (Fig. 4).

**Figure 5:**
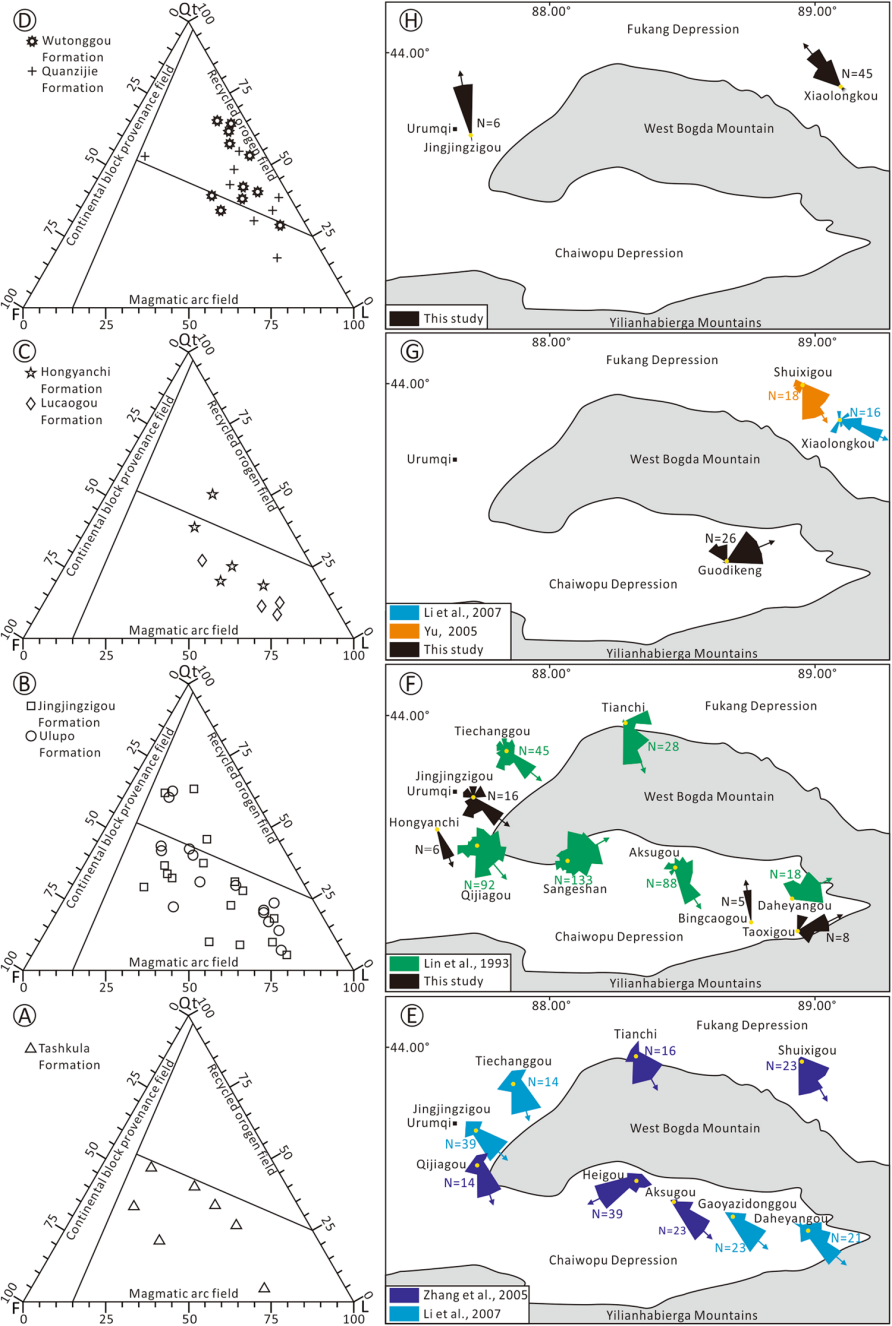
Changes of sandstone compositions in the Dickinson ternary diagrams and paleocurrent directions of the Tashkula, Ulupo, Jingjingzigou, Lucaogou, Hongyanchi, Quanzijie and Wutonggou Formations in different sections around the West Bogda Mountains. (**A**) Dickinson diagram of the Tashkula Formation. (**B**) Dickinson diagram of the Ulupo and Jingjingzigou Formations. (**C**) Dickinson diagram of the Lucaogou and Hongyanchi Formations. (**D**) Dickinson diagram of the Quanzijie and Wutonggou Formations, (**E**) Paleocurrent directions of the Tashkula Formation (data from Zhang *et al*.^50^ and Li *et al*.^47^). (**B**) Paleocurrent directions of the Ulupo and Jingjingzigou Formations (with some data from Lin^38^), (**C**) Paleocurrent directions of the Lucaogou and Hongyanchi Formations (some data from Yu^45^ and Li *et al*.^47^) and (**D**) Paleocurrent directions of the Quanzijie and Wutonggou Formations. The maps were generated by GeoMap 3.6 (http://jurassic.com.cn:8080/english/) and CorelDraw X7 (http://www.corel.com/cn/).

Petrographic analysis (thin section point counting) reveals that all the sandstone samples from the Tashkula Formation fall in the magmatic arc domain on the Dickinson diagram^34^ (Fig. 5A). Most samples of the Ulupo, Jingjingzigou, Lucaogou and Hongyanchi formations distribute in the magmatic arc domain in the Dickinson diagram (Fig. 5B,C). Most samples of the Quanzijie and Wutonggou formations fall in the recycled orogen domain (Fig. 5D).

### Stratigraphic distribution and paleocurrent direction

**Figure 6:**
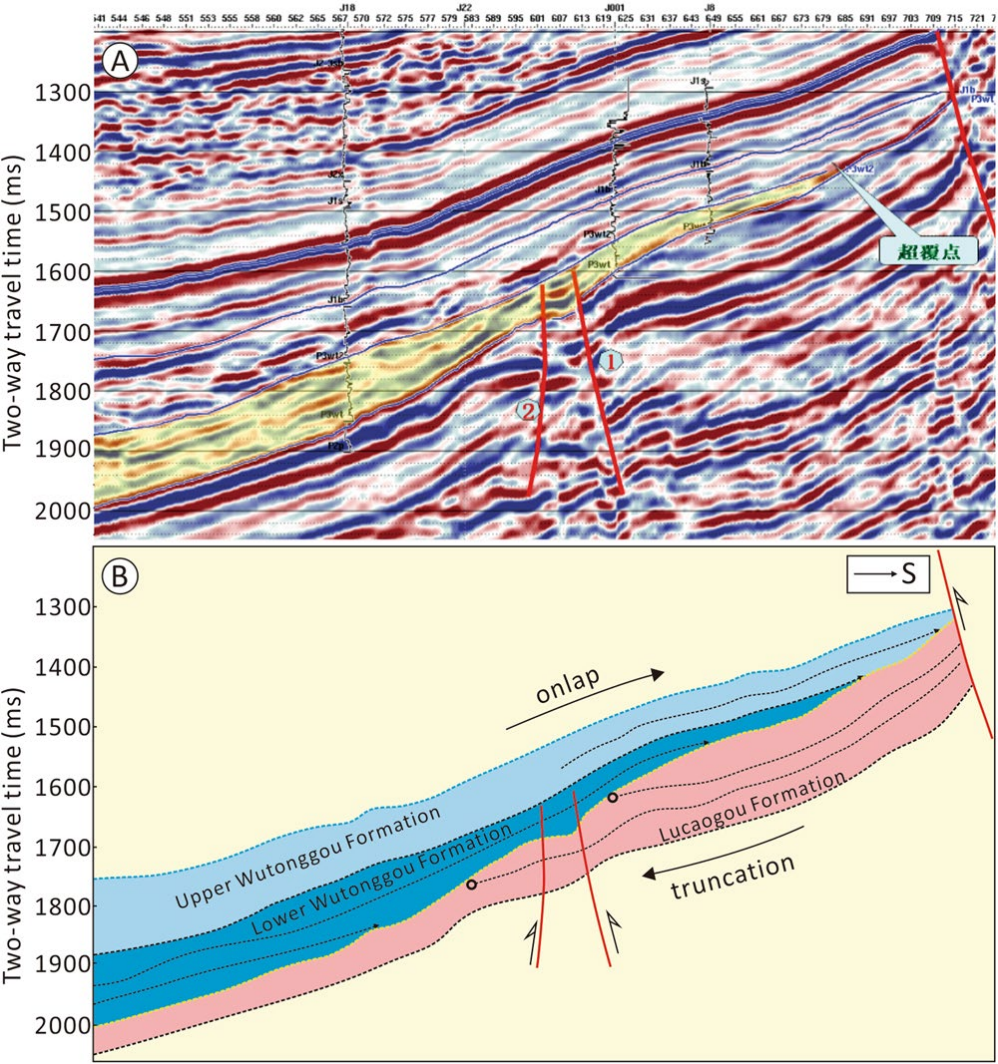
Seismic profile and illustration of the stratigraphic contact relationship between the Middle and the Upper Permian in the northern side of the West Bogda Mountains. See Fig. 1C for location.

Seismic reflection profiles from the north side of the West Bogda Mountains show that the Middle Permian sequence was truncated by the Upper Permian sequence at the downdip direction, and the Middle Permian sequence thins gradually from south to north (Fig. 6). The Upper Permian sequence overlap the Middle Permian sequence with the Upper Permian sequence thinning gradually from north to south (Fig. 6). Information on the paleocurrent directions were primarily derived from the water-flow indicators including cross beddings and current ripples recorded in the sedimentary sequences. The paleocurrent directions of the Tashkula Formation in the north and south sides of the West Bogda Mountains were mainly from NW to the SE (Fig. 5E). At the northern and western sections, and the southern Aksugou section the paleocurrent directions of the Ulupo and Jingjingzigou formations were also from NW to SE, while at the southern Sangeshan, Bingcaogou, Daheyangou and the Taoxigou sections, the paleocurrent directions were mainly from the SW to the NE (Fig. 5F). The paleocurrent directions of the Lucaogou and Hongyanchi formations in the northern Shuixigou and Xiaolongkou sections were from NW to SE, while at the southern Guodikeng section the paleocurrent direction was from SW to NE (Fig. 5G). The paleocurrent directions of the Quanzijie and Wutonggou formations in the northern Xiaolongkou and the western Jingjingzigou sections were mainly from SE to NW (Fig. 5H).

### Dolomite oxygen and carbon isotopic compositions

**Figure 7:**
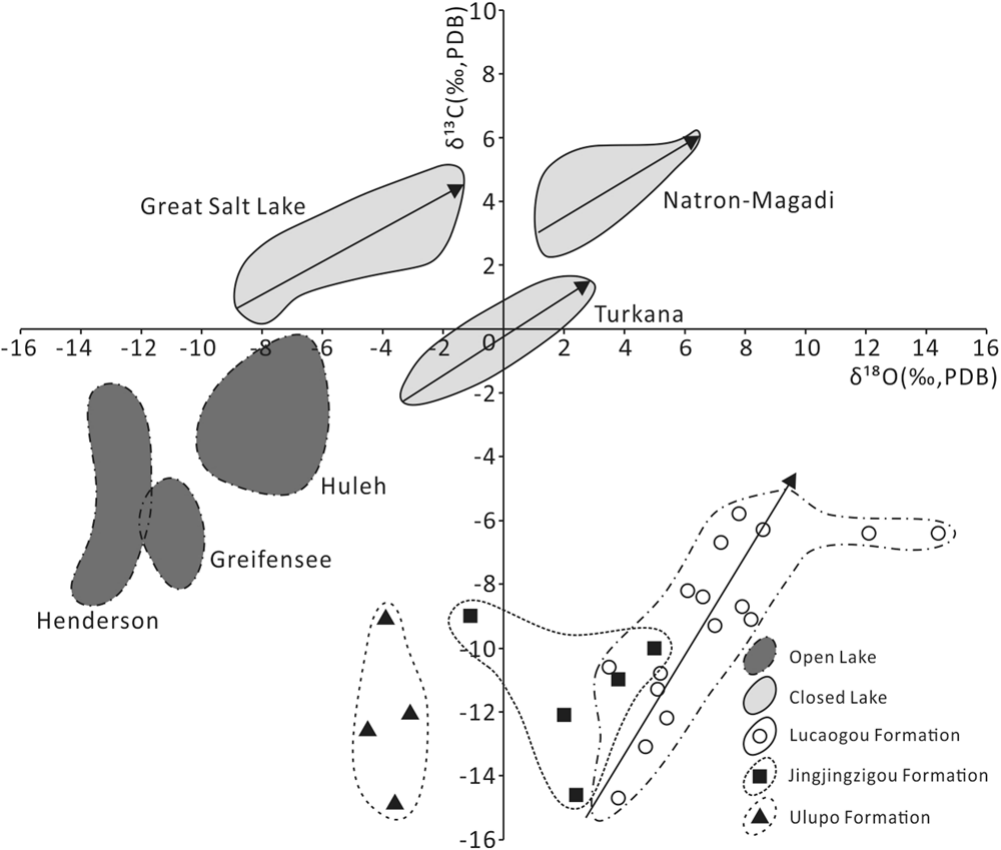
Distribution of oxygen and carbon isotopic compositions of dolomite of the Ulupo Formation, Jingjingzigou Formation and Lucaogou Formation from the sections around the West Bogda Mountains. A good linear relationship between carbon and oxygen isotope ratios occurs in the carbonate rocks from closed lake (arrows). The isotopic ratio ranges of open and closed lakes are from Talbot^41^.

The oxygen isotopic compositions (δ^18^O_PDB_) of dolomite in the Ulupo Formation is relatively light, ranging from −3.1‰ to −4.5‰, with an average of −3.8‰, whereas their corresponding carbon isotopic compositions (δ^13^C_PDB_) are between −9‰ and −14.9‰, with an average of −12.1‰. The oxygen isotopic compositions (δ^18^O_PDB_) of dolomite in the Jingjingzigou Formation is slightly heavier, ranging from −1.1‰ to 5‰, with an average of 2.4‰, whereas their corresponding carbon isotopic compositions (δ^13^C_PDB_) range from −9‰ and −14.6‰, with an average of −11.3‰. The oxygen isotopic compositions (δ^18^O_PDB_) of dolomite in the Lucaogou Formation is the heaviest, ranging from 3.5‰ to 14.4‰, with an average of 7.1‰, whereas their corresponding carbon isotopic compositions (δ^13^C_PDB_) range from −5.8‰ and −14.7‰, with an average of −9.3‰. From the Ulupo Formation to the Lucaogou Formation the oxygen isotopic compositions of dolomite become progressively heavier, their corresponding carbon isotopic compositions become slightly heavier (Fig. 7). The carbon and oxygen isotopes of the Ulupo and Jingjingzigou formations are not apparently correlated, while the carbon and oxygen isotopes of the Lucaogou Formation are relatively well correlated (Fig. 7).

## Discussion

### Rapid change in depositional environments

The depositional environment of the Permian at the west Bogda area experienced a complicated evolution. The Early Permian palaeogeographic configurations of the west Bogda area inherited that of the Carboniferous period, which was an important crustal rifting stage^11,12,15^. Strong crustal expansion formed a long and narrow ocean trough (deep-water basin), dominated by a slope, semi-deep to deep marine environment^11^. At the Early Permian, the expansion of the West Bogda Trough reached its pinnacle, the water depth was greatly increased, and the Shirenzigou Formation was deposited as a series of slumping coarse clastic rocks with interlayers of pillow lava-vesicular basalt^11^. *Brachiopods*, *bivalves* and *Crinoid stems fossils* were widely present in the sediments^33^. Combined with the sedimentary characteristics, the Shirenzigou Formation was dominated by gravity flow deposits at shelf-slope settings^35,36^. The lithology of the Shirenzigou Formation is mainly conglomerate and pebbly sandstone, and the gravels consist mainly of basaltic andesitic porphyrite from the Late Carboniferous Liushugou Formation, indicating the provenance is mainly from the northern Santai-Qitai area (see Fig. 1B for location)^36^. The deposits of the Lower Tashkula Formation consist mainly of frequent interlayers of argillaceous siltstone and mudstone with a small amount of sandstones, belonging to normal shelf deposition^28,37^. Groove structures of erosion origin and deep water trace fossils at the base of the sandstone suggest that they were deposited as turbidites in a deep water environment^36,37^. The Middle-Upper Tashkula Formation is characterized by interlayers of deep water mudstone and medium-thick sandstone with hummocky-sunken cross beddings (Fig. 3 and Supplementary Fig. S1B–C), which are typical of storm deposits in a deep-water high-energy environment^38^. At the end of the Early Permian, affected by the collision of the southern Tarim Block and the northern Junggar Block (Siberia Plate), the east Bogda Trough was closed and uplifted, leading to the closure of the west Bogda Trough^8,12,17,21,23,36^. The presence of shallow water sandstones of the Ulupo Formation directly overlying on the deep water mudstones of the Tashkula Formation indicates a rapid depositional environment change, which also attests the closure of the Bogda Trough and the basement uplifting at the end of the Early Permian.

The closure and subsequent basement uplifting of the West Bogda Trough caused the seawater gradually withdrew and the depositional environment change from marine setting to a closed epicontinental offshore lacustrine setting with shallow water during the early stage of the Middle Permian^24,33,35,39^. Changes in fossil assemblages also reflect the change of sedimentary environments from marine to nonmarine settings at the early stage of the Middle Permian^33^. Frequent interlayers of mudstone and sandstone with abundant sedimentary structures, including wave ripple, ripple cross bedding and herringbone cross bedding (Supplementary Fig. S1D–F), indicate that the depositional environment of the Ulupo Formation was likely of a tidal flat setting^35,37^. The extensively developed, normally graded bedding with scour surfaces at bases, parallel bedding, and arranged gravels with sub-rounded shape (Supplementary Fig. S1G–H) and reversely graded sandstone indicate that the Jingjingzigou Formation was dominated by a shallow water environment and braided delta. During the depositional period of the Lucaogou and Hongyanchi formations, the basement subsided, and the deposition of interbeds of thick layered gray-dark shale and medium-thin layers of dolomite and dolomitic shale (Supplementary Fig. S2A–D, I–J). The fossil assemblages suggest that the depositional environment gradually changed into a semi-deep to deep lacustrine environment. Compared to the stages of the Ulupo Formation and Jingjingzigou formations, the lake size expanded and lake level rose. At the end of the depositional period of the Hongyanchi Formation the lake level fell and a shore-to-shallow lacustrine environment once again became dominated owing to a slow regional tectonic uplift^37^. Carbonate rocks older than the Jurassic can best be classified into marine and fresh-water origins by referencing to the carbon isotope ratios alone^40^. The ranges of carbon isotope ratios of the Ulupo, Jingjingzigou and Lucaogou formations are in the range of the Permian fresh-water carbonates (δ^13^C_PDB_ = −0.4‰ to −11.06‰)^40^, suggesting a typical lacustrine fresh-water setting during the Middle Permian. The progressively heavier oxygen isotopic compositions of the dolomite from the Ulupo Formation to the Lucaogou Formation, and the relatively good linear relationship between carbon and oxygen isotope ratios of the Lucaogou Formation (Fig. 7) indicate closure of the depositional environment due to a sustained basal subsidence^41^, which suggests that the depositional environment had not changed rapidly but evolved gradually from a shallow, to a deep and then to a shallow lacustrine setting during the Mid-Permian.

The deposits of the Late Permian Quanzijie and Wutonggou formations show obvious near-source sedimentary characteristics and are characterized by purplish-reddish conglomerate and pebbly sandstone with extremely poorly-sorted and rounded gravels (Supplementary Fig. S3). They are typical of molasse formations, illustrating a rapid change of the depositional environment compared to the Middle Permian^19,42,43^. The Late Permian molasse belts mainly distribute around the West Bogda Mountains, indicating that the depositional process were closely connected with the uplift of the West Bogda Mountains. The rapid depositional environment change and the features and distribution of the molasse belts both suggest a significant uplift of the West Bogda Mountains at the end of the Middle Permian.

### Abrupt changes in sediment provenances

Strong orogeny can usually change the paleogeographic configurations of the earth’s surface, which are generally embodied in the transformations of the provenance areas of a basin and the source rock properties^34^. The provenance area of the Lower Permian was mainly a magmatic arc according to Dickson’s template (Fig. 5A). Gravels in conglomerate and pebbly sandstone are mainly of igneous rocks with modest sorting and roundness (Table 1), indicating a relatively long-distance transport from the source to sink. The provenance property of the Middle Permian primarily inherited from the Lower Permian as indicated by the gravel types and contents and Dickinson diagram. The provenance area of the Middle Permian was also dominated by a magmatic arc (Fig. 5B,C). Although the West Bogda Trough was closed and the depositional environment changed from marine to a closed epicontinental offshore lake^12,17,21,36^, the inherited provenance property indicates that the denudation patterns and the paleogeographic configuration had not changed much from the Lower to the Middle Permian. The provenance area and the source rock property of the Upper Permian, however, were transformed distinctly according to the gravel types and contents and Dickinson diagram (Table 1 and Fig. 5D). The provenance area of the Late Permian was mainly of recycled orogenic rocks with a small amount of magmatic arc in origin (Fig. 5D). The gravels in the Upper Permian deposits have extremely poor sorting and roundness (Fig. 6). Gravels of sedimentary rocks increase distinctly, including gravels of sandstone, siltstone, dolomitic shale, dolomite and gray-dark shale (Supplementary Fig. S3 and Table 1). In the study area, only the Middle Permian Lucaogou Formation and the Hongyanchi Formation comprise interlayers of gray-dark shale, dolomitic shale, dolomitic siltstone and dolomite. This suggests that the deposits of the Upper Permian were mainly derived from the Middle Permian Lucaogou and Hongyanchi formations. XRD analysis shows that the mineral compositions of different gravels of sedimentary origin in the Upper Permian correlated well with the corresponding rocks of the Middle Permian Lucaogou and Hongyanchi formations (Fig. 4). This further confirms that the provenance of the Upper Permian was mainly from the Middle Permian Lucaogou and Hongyanchi formations. The abrupt change of the provenance area and the source rock property of the Upper Permian, and the presence of recycled Middle Permian sedimentary rocks in the Upper Permian sequence suggest that the Middle Permian or older formations must have been uplifted and denuded. Therefore a significant uplift of the West Bogda Mountains may have occurred at the end of the Middle Permian. The increasing contents of igneous rock and tuff gravels from the Quanzijie to Wutonggou formations also suggested a sustained uplift of the West Bogda Mountains during the Late Permian. Because the sustained uplift of the West Bogda Mountains caused the decrease of sedimentary rocks at the denuded zone and the increase of denuded amount of Carboniferous rocks.

### Migration of sedimentary and subsidence centers

Paleocurrent analysis is an important part of the basin analysis, which can be used to infer the regional paleogeomorphology, provenance direction and sedimentary environment^44^. At the north, west and the south sections around the West Bogda Mountains, the paleocurrent directions were almost entirely from NW to SE during the depositional period of the Lower Permian Tashkula Formation (Fig. 5E). This suggests that the west Bogda area was an underwater slope and the sedimentary and subsidence center of the basin were further south at this stage^37,45^. The Middle Permian paleocurrent directions at the north and west of the west Bogda area inherited that of the Early Permian (Fig. 5F,G). The paleocurrent directions at the south of the west Bogda area were mainly from SW to NE except for the Aksugou section, where the paleocurrent direction was from NW to SE (Fig. 5F,G). The change of the paleocurrent direction in the south of the west Bogda area also suggests that the west Bogda area were still located in the catchment area and the sedimentary and subsidence center gradually moved northward^19,45^. In contrast to the Early and Middle Permian, the paleocurrent directions at the north and west of the west Bogda area were from SE to NW during the Late Permian (Fig. 5H), which suggests that the west Bogda area was uplifted, and the previous catchment area was switched to become a denudation area. The originally unified sedimentary and subsidence center was divided into the north Fukang Depression and the south Chaiwopu Depression (see Fig. 1B for location). The presence of reversed Middle Permian formations and the inconsistency of the formation thickness (Fig. 6) also indicate a distinct transformation of the paleogeographic configuration from the Middle Permian to the Late Permian. The west Bogda Mountains were uplifted, which caused the stratigraphic reversal of the Middle Permian stratum at the end of the Middle Permian.

### Initial uplift process of the West Bogda Mountains

**Figure 8:**
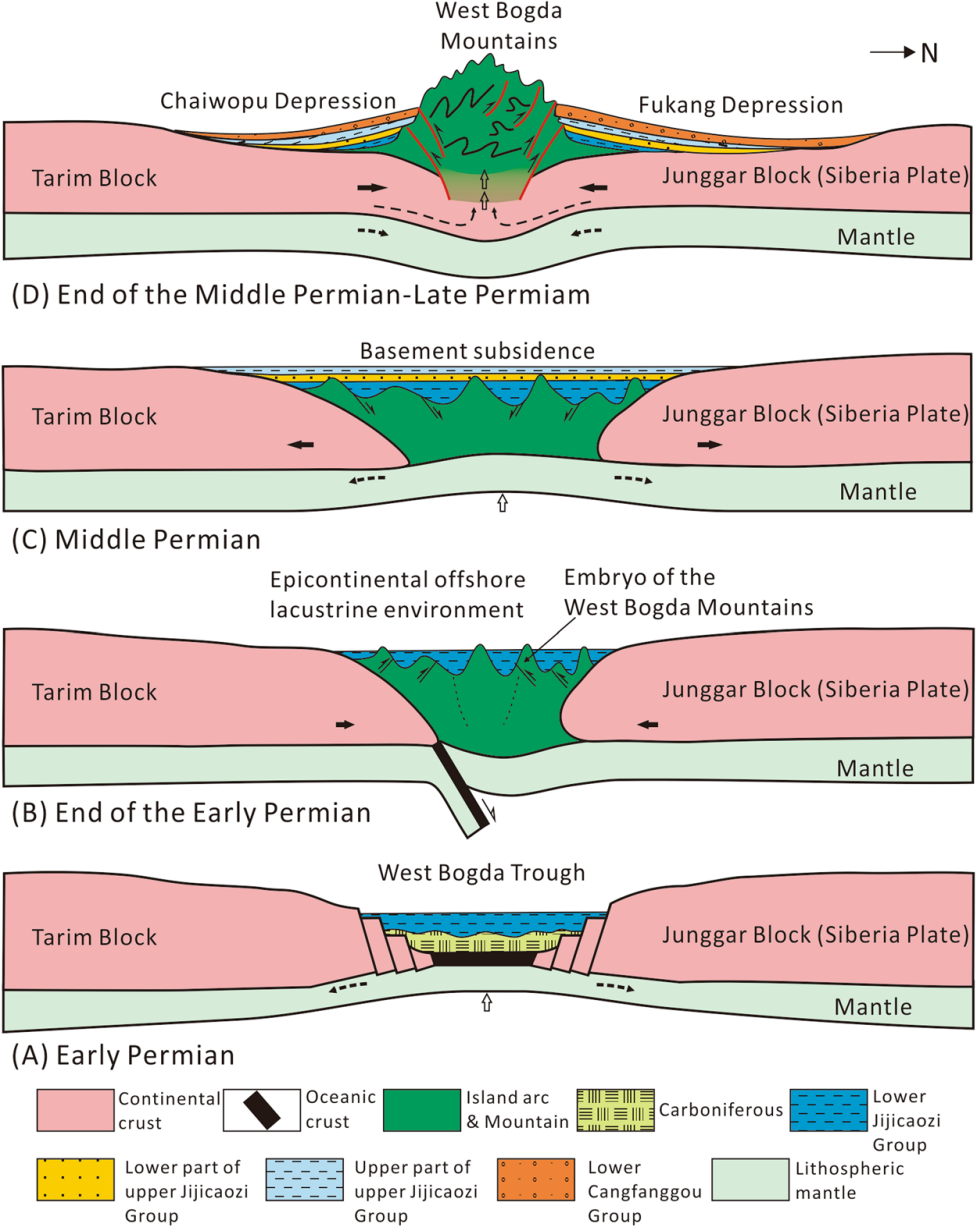
Evolutionary model of the initial uplift process of the West Bogda Mountains at the end of the Mid-Permian.

For a long time, there have been mainly two schools of thought on the Late Paleozoic tectonic attributes of the Bogda Mountains. The Bogda Mountains were either regarded as an island arc formed in the Carboniferous by some, or as a rift basin by others. However, recently, two epochs of bimodal volcano rocks of the Carboniferous and Early Permian were recognized in the Bogda Mountains. The current prevailing view is that the Bogda Mountains was of a Carboniferous-Early Permian rift basin. A wealth of evidence including the transformation of the depositional environments, provenance area and source rock types, sedimentary and subsidence centers, and development of bimodal volcanic-sedimentary rock series^11,15,32^ indicates that the tectonic framework of the Early Permian inherited that of the Late Carboniferous, which was characterized by a rift basin with a series of slumping coarse clastic rocks with interlayers of pillow lava-vesicular basalt^11,19^ (Fig. 8A). At the end of the Early Permian, the Junggar-Tianshan ocean disappeared completely, the West Bogda Trough was closed with the concomitant development of an island arc as a result of the collision between the north Junggar Block (Siberia Plate) and the south Tarim Block^8,12,17,21,23,36^. The west Bogda area shifted to the intracontinental tectonic evolution stage^8,12,21,46^. The Bogda ductile shear deformation zone was formed during this process, marking the onset of the West Bogda Mountains orogeny^19^. This orogeny was initially weak and has limited impact and most of the West Bogda Mountains were still under water with only small parts emerged (Fig. 8B) probably due to the slow subduction of the oceanic crust and a weak compression^14,18,19^. This explains why the Middle Permian in this area was still within the catchment area. The orogeny created an embryo of the West Bogda Mountains and laid the foundation for the subsequent strong orogeny at the end of the Middle Permian^18,19^. The island arc region continued to receive deposits during the Middle Permian possibly due to the relaxation of the compression between the Junggar and Tarim Blocks and isostatic rebound of crustal deformation. Continued basement subsidence caused the basin in the West Bogda Mountains area gradually closed^35,37^. The depositional environment, provenance and sedimentary and subsidence centers display good inheritance characteristics from the Early Permian to the Middle Permian (Fig. 8C). Strong intracontinental collision occurred between the north Junggar Block and the south Tarim Block at the end of the Middle Permian, causing the West Bogda Mountains uplifted. The uplift of the West Bogda Mountains caused abrupt changes in the depositional environment, provenance and sedimentary and subsidence centers from the Middle Permian to the Late Permian (Fig. 8D).

Sedimentological evidence further confirmed that the area of the West Bogda Mountains was a rift basin in the Carboniferous-Early Permian. Subsequently it entered the Late Paleozoic large-scale intracontinental orogeny, Mesozoic peneplanation and Cenozoic revival orogeny after a brief stable epicontinental basinal environment during the Middle Permian^10,13,16,20,47^. Because of the unique tectonic location of the West Bogda Mountains, the Permian tectonic and sedimentary evolution of the west Bogda area provides some direct indication of the tectonic attribute and evolution of the west part of CAOB in the Late Paleozoic. The Late Paleozoic large scale orogenic movement in the west part of CAOB was probably no earlier than the Middle Permian. Because the typical intracontinental revival orogenic belt was far from plate boundaries, the Late Paleozoic evolution of the Tianshan orogenic belt provides a good reference for the evolution of other orogenic belts with similar tectonic settings.

## Conclusions

On the basis of a detailed investigation of outcrops including analyses of lithology, sedimentary structures, mineral and isotopic compositions, and paleocurrent directions, this study provides new insights into the tectonic and sedimentary evolution of the west Bogda area and the timing of initial uplift of the West Bogda Mountains:

1. At the end of the Early Permian, the West Bogda Trough was closed and an island arc was formed owing to the collision between the north Junggar Block and the south Tarim Block, which marked the beginning of the West Bogda Mountains orogeny and created the embryo of the West Bogda Mountains. Most of the West Bogda Mountains were still under water with small portions being un-inundated probably due to the slow subduction of the oceanic crust and a relatively weak compression. Sediment deposition continued in the Bogda area during the Middle Permian.
2. During the Middle Permian, the Bogda area gradually became a more closed depositional setting because of the basement subsidence. The depositional environment, source and sink centers had good inheritance characteristics from the Early to the Middle Permian. The west Bogda area continued to receive deposits and developed a widespread shallow, deep and then shallow lacustrine succession during the Mid-Permian.
3. At the end of the Middle Permian, a strong intracontinental collision caused uplift of the West Bogda Mountains, which separated the Fukang Depression from the Chaiwopu Depression. The early strata were uplifted and denuded to provide the sediment source for the Late Permian deposits effected by the uplift of the West Bogda Mountains. Sedimentological evidence further confirmed that the West Bogda Mountains was a rift basin in the Carboniferous to Early Permian, and subsequently entered the Late Paleozoic large-scale intracontinental orogeny, which formed the west part of CAOB probably no earlier than the Middle Permian.

## Materials and Analytical Methods

### Outcrop sections and sampling positions

Six Permian outcrop sections around the West Bogda Mountains were investigated in detail from the Tashkula Formation to the Wutonggou Formation including: the Xiaolongkou section, Xidalongkou section, Jingjingzigou section, Hongyanchi section, Guodikeng section and Bingcaogou section (see Fig. 1C for locations of sections). The detailed lithology and sedimentary structures of the six sections were studied as well as their stratigraphic characteristics. The outcrops studied were sampled systematically for lithological, mineral and carbon-oxygen isotopic composition analyses (see Fig. 3 for sampling positions). The stratigraphic and sedimentary characteristics of the Shirenzigou Formation are mainly derived from previous studies because of the poor outcrop exposure in the studied sections.

### Rock composition analysis

Gravel types and contents were analyzed in the field and laboratory via outcrop visual inspection and thin section observation. Firstly, several 100 cm × 100 cm areas in a gravel-bearing outcrop were selected to preliminarily classify the types of gravels and count the numbers of each type and the total gravel numbers based on hand specimen identification. The total numbers of gravels in a formation counted were no less than 150. Secondly, thin sections were made for each gravel type. The gravel types and numbers were primarily determined on the basis of thin section analysis.

A total of 66 sandstone samples were analysed. Compositional modal analyses of thin sections were performed by counting 200 points per thin section. The classification scheme for sandstone of Folk^48^ was used. Twenty-one samples were collected for mineralogical analyses using X-ray diffraction (XRD). A D8 DISCOVER was used for XRD analysis with Cu-Ka radiation, a voltage of 40 kV, and a current of 25 mA. Prior to analysis, each sample was oven-dried at 40 °C for 2 days and ground to <40 μm in sizes using an agate mortar to thoroughly disperse any aggregated minerals. No chemical pre-treatment was employed. Samples were scanned from 3° to 70° with a step size of 0.02°. Quantitative analysis of the diffractograms provided identification and semi-quantitative analysis of the relative abundance (in weight percent) of the various mineral phases.

### Carbon and oxygen isotopic analysis

Twenty-five dolomite samples selected were first ground below 200 mesh and then filtered through a 325 mesh sieve. The grains used for analysis were mostly approximately 5 μm–44 μm. CO_2_ was extracted using the stepwise reaction method developed by Al-Aasm *et al*.^49^, which consists of the following steps: (1) Samples were dried at 60 °C for 12 h and baked at 110 °C for 3 h; (2) Approximately 150 mg of the sample was weighed and transferred into the main reaction tube, and a suction pipette was used to deliver 2.5 mL of anhydrous phosphoric acid to a branch tube; (3) The sample was dehydrated for 1 h–2 h in a vacuum higher than 2 Pa; (4) The sample was allowed to react with the phosphoric acid for 1 day in a water-bath (thermostatic chamber) at 50 °C and the CO_2_ released from dolomite was then extracted. Carbon and oxygen isotopes of CO_2_ were analyzed using a Finnigan Mat 250 mass spectrometer. The Peedee Belemnite (PDB) standard was used for the carbon isotope analysis, while the Standard Mean Ocean Water (SMOW) standard was used for the oxygen isotope analysis. The precisions of the carbon and oxygen isotope ratios were ± 0.2‰ and ± 0.3‰, respectively. Oxygen isotope ratios referencing to the PDB standard were calculated using δ^18^O_v-SMOW_ = 1.03086 × δ^18^O_v-PDB_ + 30.86 of Friedman and O’ Neil^49^.

### Paleocurrent direction analysis

The paleocurrent directions of different formations were determined on the basis of the detailed study of stratigraphic characteristics and sedimentary structures. The procedure was as follows: (1) selecting the sedimentary structures that reflects the direction of a unidirectional flow as the measurement objects, including cross beddings with foreset laminae (such as tabular cross bedding, wedge-shaped cross bedding, ripple cross bedding), arranged gravels and asymmetrical ripples, to determine their dips and dip angles. At least five groups of data were measured at a measuring point. (2) The dips and dip angles of the target strata were determined. (3) The original occurrence conditions of primary sedimentary structures were restored by using the stereographic projection method based on the measuring data of step (1) and (2)^44^. (4) The paleocurrent rose-diagram for every formation was plotted^44^. The azimuth was spaced at intervals of 10 degrees and divided into 36 groups for sorting analysis.

### Data Availability

The datasets generated during and/or analysed during the current study are available from the corresponding author on reasonable request.

## Electronic supplementary material

Supplementary Information
