# Supplementary material for: Sedimentological constraints on the initial uplift of the West Bogda Mountains in Mid-Permian
Source: Sci Rep. 2018 Jan 23;8:1453. doi: 10.1038/s41598-018-19856-3 (PMC5780466; doi:10.1038/s41598-018-19856-3)
Supplement: Supplementary file 1 — Supplementary Information [file 41598_2018_19856_MOESM1_ESM.doc]

**Sedimentological constraints on the initial uplift of the West Bogda Mountains in Mid-Permian**

Jian Wang^1, 2 *^, Ying-chang Cao^1, 2^, Xin-tong Wang^1^, Ke-yu Liu^1, 3^, Zhu-kun Wang^1^ & Qi-song Xu^1^

^1^ *School of Geosciences, China University of Petroleum (East China), Qingdao 266580, China*

^2^ *Laboratory for Marine Mineral Resources, Qingdao National Laboratory for Marine Science and Technology, Qingdao, 266071, China*

^3^ *Department of Applied Geology, Curtin University, GPO Box U1987, Perth, WA 6845, Australia*


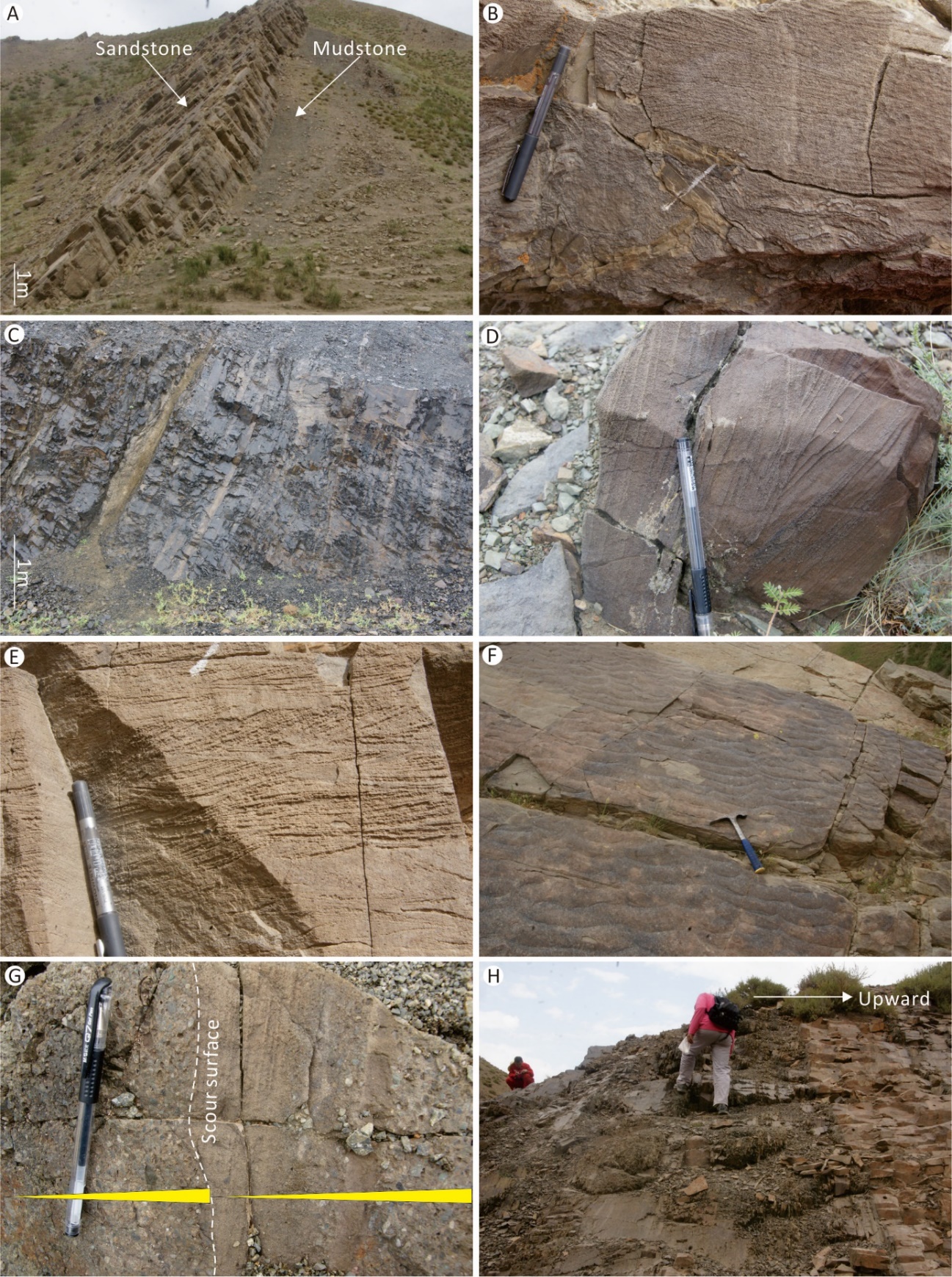


**Figure S1.** Typical sedimentary characteristics of the Permian Tashkula, Ulupo and Jingjingzigou Formations. (A) Normally graded sandstone in contact with gray mudstone, Tashkula Formation, Jingjingzigou section. (B) Hummocky-sunken cross bedding in sandstone, Tashkula Formation, Jingjingzigou section. (C) Thick layers of dark-gray mudstones interbedded with thin layers of gray siltstones, Tashkula Formation, Hongyanchi section. (D) Herringbone cross beddings in sandstones, Ulupo Formation, Hongyanchi section. (E) Wave ripple cross beddings in fine sandstones, Ulupo Formation, Jingjingzigou section. (F) Symmetric wave ripples in fine sandstones, Ulupo Formation, Jingjingzigou section. (G) Normally graded cycles of pebbly sandstone-fine sandstone-siltstone with scour surfaces, with gravels comprising mainly igneous rocks with a certain degree of sorting and rounding, Jingjingzigou Formation, Hongyanchi section. (H) Reverse graded sequence with gray mudstone-siltstone-fine sandstone, Jingjingzigou Formation, Jingjingzougou section.

**
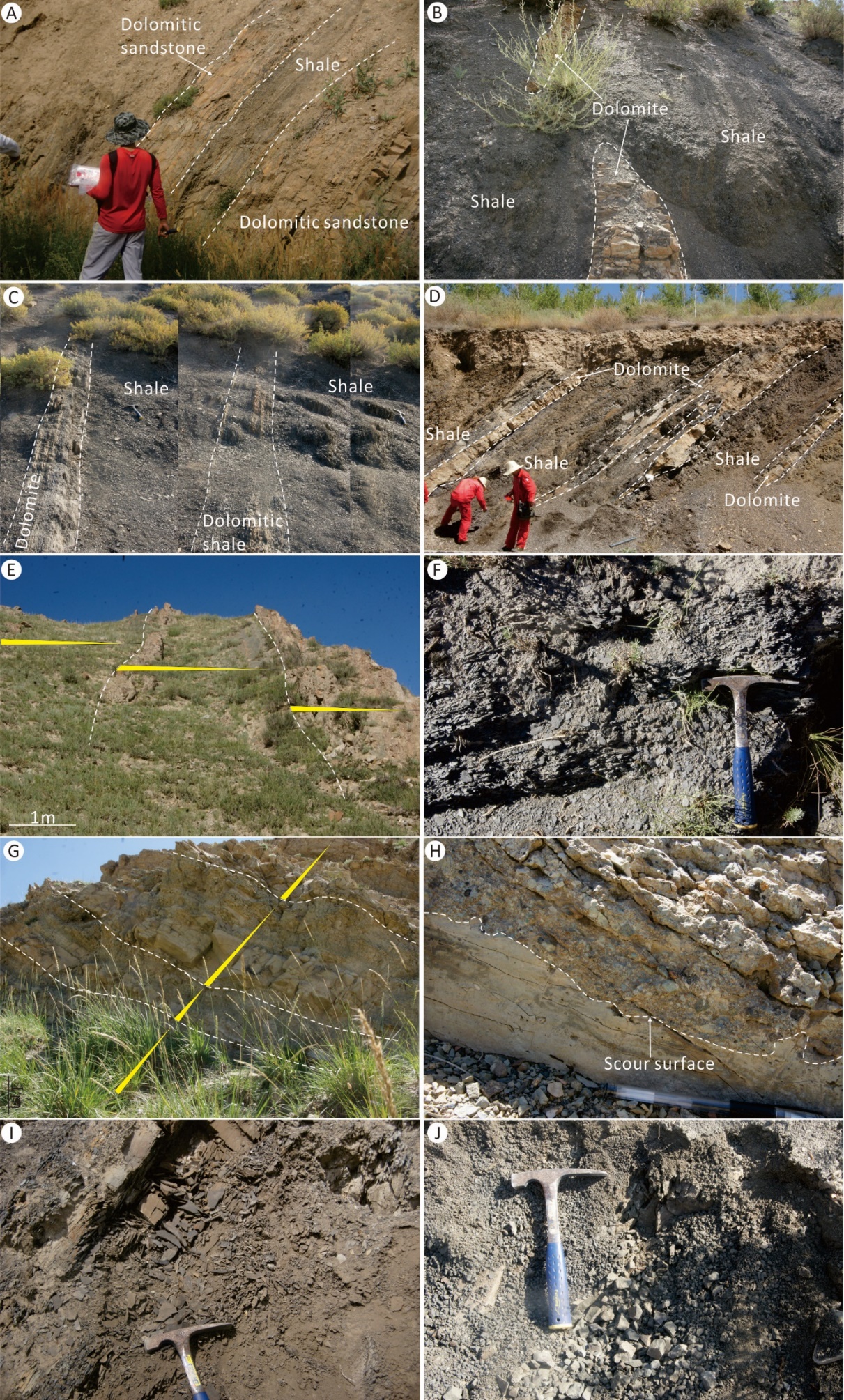
**

**Figure S2.** Typical sedimentary characteristics of the Permian Lucaogou and Hongyanchi Formations. (A) Dolomitic sandstones interbedded with gray-dark shale, Lucaogou Formation, Xiaolongkou section. (B) Gray-dark shale with lenticular dolomites, Lucaogou Formation, Xidalongkou section. (C) Gray-dark shale with thin interlayers of dolomites and dolomitic shales, Lucaogou Formation, Jingjingzigou section. (D) Gray-dark shale with thin interlayers of dolomites, Lucaogou Formation, Hongyanchi section. (E) Thick layers of normal grain order cycles of gray pebbly sandstone-fine sandstone-gray-dark mudstone with scour surface, Lucaogou Formation, Guodikeng section. (F) Gray-dark shale at the top of the normal grain order cycles, Lucaogou Formation, Guodikeng section. (G) Thick layers of normal grain order cycles of gray pebbly sandstone-fine sandstone-dark-gray mudstone with scour surface, Lucaogou Formation, Bingcaogou section. (H) Scour surface at the bottom of the pebbly sandstone in the normally graded cycles with gravels mainly of igneous rocks with modest sorting and rounding, Lucaogou Formation, Bingcaogou section. (I) Gray-dark shale, Hongyanchi Formation, Xiaolongkou section. (J) Gray-dark shale, Hongyanchi Formation, Jingjingzigou section.

**
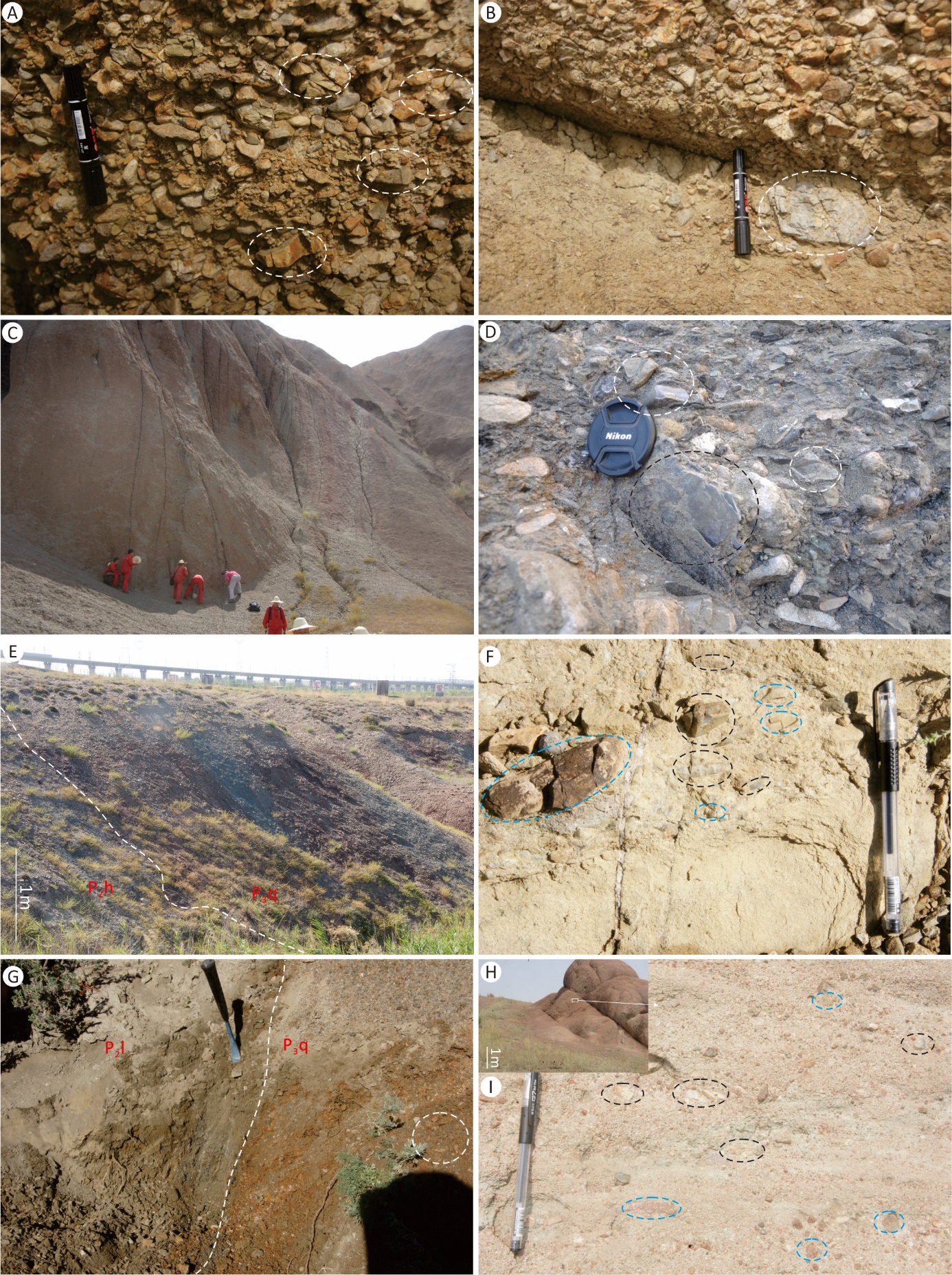
**

**Figure S3.** Typical sedimentary characteristics of the Permian Quanzijie and Wutonggou formations. (A) Conglomerates with extremely poorly sorted and rounded gravels with frequent presence of dolomitic gravels (white circles), Quanzijie Formation, Xiaolongkou section. (B) Conglomerates with extremely poorly sorted and rounded gravels and dolomitic gravels (white circle), Quanzijie Formation, Xiaolongkou section. (C) Purplish-reddish conglomerates, Quanzijie Formation, Xidalongkou section. (D) Conglomerates with extremely poorly sorted and rounded gravels and dolomitic gravels (white circle) and dark-grayish shale gravels (black circle), Quanzijie Formation, Xiaolongkou section. (E) The contact between the Hongyanchi Formation with gray-dark shale and the Quanzijie Formation with purplish-reddish conglomerates, Hongyanchi section. (F) Conglomerates with extremely poorly sorted and rounded gravels and dolomitic gravels (black circle) and sandstone gravels (blue circle), Quanzijie Formation, Hongyanchi section. (G) The contact between the Lucaogou Formation with dark-gray shale and the Quanzijie Formation with purplish-reddish conglomerates, Guodikeng section. (H) and (I) Purplish-reddish conglomerates with extremely poorly sorted and rounded gravels and dolomite gravels (black circle) and sandstone gravels (blue circle), Wutonggou Formation, Jingjingzigou section.
